# Supplementary material for: A flexible approach for variable selection in large-scale healthcare database studies with missing covariate and outcome data
Source: BMC Med Res Methodol. 2022 May 4;22:132. doi: 10.1186/s12874-022-01608-7 (PMC9066834; doi:10.1186/s12874-022-01608-7)
Supplement: Supplementary file 1 — Additional file 1 Web-based supplementary materials. Supplementary Section 1. Random Forest based Imputation Algorithm - missForest. Supplementary Section 2. Simulation Setup. Supplementary Section 3. Additional tables (Supplementary Table 1–12) and figures (Supplementary Figure 1–2) for simulation study and case study. [file 12874_2022_1608_MOESM1_ESM.docx]

**Web-based Supplementary Materials for “A flexible approach for variable selection in large-scale healthcare database studies with missing covariate and outcome data''**

Jung-Yi Joyce Lin, Liangyuan Hu*, Chuyue Huang, Jiayi Ji, Steven Lawrence and Usha Govindarajulu

**S1. Random Forest based Imputation Algorithm - missForest**

Proposed by Stekhoven and Buhlmann ^2^, missForest is a non-parametric imputation method using Random Forest. The missForest proceeds as follows. Initial guesses are made for the missing values (e.g., mean or mode) , and variables are sorted in the ascending order of missingness proportions. The variables with missing data are in turn, in the descending order of missingness proportions, regressed via Random Forest on other variables. The missing values are imputed/updated using the fitted Random Forest model as the prediction model. This process is repeated until a stopping criterion is met or the maximum number of iterations is reached. After each iteration, the differences for continuous variables **N** and categorical variables **F** between the newly imputed data and the previous data will be calculated as

$$\Delta N=\frac{\sum_{j\epsilon N} {(X_{new}^{imp}-X_{old}^{imp})}^{2}}{\sum_{j\epsilon N} {(X_{new}^{imp})}^{2}}$$

$$\Delta F=\frac{\sum_{j\epsilon F} \sum_{i=1}^{n} I_{X_{new}^{imp}\neq X_{old}^{imp}}}{\# of missing values in F}$$

The stopping criterion is met as soon as both differences increase for the first time.

Stekhoven and Buhlmann ^2^ showed that missForest produced more accurate imputation than MICE because of the improved flexibility of the imputation model offered by Random Forest. Additionally, Hu, et al. ^3^ showed the variable selection methods performed better on incomplete data when combined with missForest than combined with MICE. Thus, we used missForest for the imputation.

We used default values for the tuning parameters of the missForest R function. Specifically, ntree=100, mtry=$\sqrt{K}$ (total number of predictors), nodesize=5 for categorical variables and 1 ﻿for continuous variables, and maxnodes=NULL.

**S2. Simulation Setup**

First consider the sample size $n=1000.$We generated 10 useful variables and 40 noise variables. The distribution of the variables is described below.

$$x_{1}, x_{2} \sim Bern(0.5)$$

$$x_{3}, x_{4} \sim N(0,1)$$

$$x_{5} \sim Gamma(4, 6)$$

$$x_{7}|x_{5}, x_{6} \sim N(-0.4x_{5}+0.4x_{6}+0.3x_{5}x_{6}, 1)$$

$$x_{8}|x_{5}, x_{6}, x_{7} \sim N(0.1x_{5}\left( x_{6}-2 \right)^{2}-0.1{x_{7}}^{2}, 1)$$

$$x_{9}|x_{3}, x_{4},x_{5} \sim N(0.5x_{3}+0.3x_{4}-0.3{x_{5}}^{2}+0.2x_{3}x_{4}, 1)$$

$$x_{10}|x_{3}, x_{4},x_{5}, x_{9}\sim N(0.1{x_{3}}^{2}-0.3x_{4}-0.4x_{5}+0.2{x_{9}}^{2}+0.3x_{5}x_{6}, 1)$$

$$\Pr\left( y=1 | x_{1}, \ldots, x_{10} \right)={logit}^{-1}(=2.7+1.8x_{1}+0.5x_{2}+1.1x_{3}-0.4e^{x_{5}}-\left( x_{6}-3.5 \right)^{2}+0.3\left( x_{7}-1 \right)^{3}+1.1x_{8}-1.1x_{10}+5\sin\left( 0.1\pi x_{4}x_{9} \right)-0.4{{x_{5}x}_{10}}^{2}+0.4{x_{3}}^{2}x_{8})$$

After generating the full data, we amputed $X_{7}$, $X_{8}$, $X_{9}$, $X_{10}$, and *Y* using the multivariate amputation approach. ^1^ We first considered 40% missingness in *Y* and 60% overall missingness. To ampute the data, we first randomly divided the full data into 8 subsets with the following percentages of the whole data: 0.30, 0.09, 0.09, 0.08, 0.08, 0.16, 0.10, 0.10, and then amputed 60% of data points in each subset. Each subset was amputed based on the weighted sum scores, which relate the missingness on amputated variables to the values of other variables. To create a missing at random (MAR) variable, a zero weight was assigned to the variable itself. The weighted sum scores in this simulation were set as below:

1. ${wss}_{y,i}=5x_{1}+5x_{2}+x_{3}-x_{5}-x_{6}+x_{7}+x_{8}+x_{10}-0.5{x_{6}}^{2}+1.5x_{4}x_{9}-0.5x_{5}x_{10}+0.5x_{3}x_{8}$
2. ${wss}_{x_{7},i}=x_{5}+x_{6}+x_{5}x_{6}$
3. ${wss}_{x_{8},i}={5y+x}_{5}+x_{6}+x_{7}+{x_{7}}^{2}+x_{5}x_{6}$
4. ${wss}_{x_{9},i}={5y+x}_{3}+x_{4}+x_{5}+{x_{5}}^{2}+x_{3}x_{4}$
5. ${wss}_{x_{10},i}={5y+x}_{3}+x_{4}+x_{5}+x_{9}+x_{4}x_{5}$
6. ${wss}_{y, x_{7},x_{8},i}=x_{5}+x_{6}$
7. ${wss}_{y,x_{8},x_{10}i}=x_{5}$
8. ${wss}_{y, x_{9}, x_{10}, i}=x_{3}+x_{4}+0.5{x_{5}}^{2}+x_{3}x_{4}$

The probability of a data point in a subset became missing or not was then calculated by applying a logistic distribution function on the weight sum scores. A right-tailed type of missingness was used for subsamples (1)–(5) and a both-tailed type of missingness was used for subsamples (6)–(8). After putting all subsets together, we had a full data set with desired missing characteristics.

To generate data with 30% overall missingness and 20% missingness in *Y*, we amputed 30% of data points in each of eight subsets. These data generating processes are repeated to simulate data with different sample sizes, $n=300, 650, 5000$. We also considered different ratios of useful versus noise predictors by keeping 10 useful predictors and generating 10 and 20 noise predictors, with half of them simulated from the standard normal distribution and half from Bern(0.5).

**S3. Additional tables and figures**

**Supplementary Table 1.** Pearson correlations between the 8 continuous variables ($X_{3},X_{4}, \ldots, X_{10}$). Ind = Independent.

|  | $X_{3}$ | $X_{4}$ | $X_{5}$ | $X_{6}$ | $X_{7}$ | $X_{8}$ | $X_{9}$ | $X_{10}$ |
| --- | --- | --- | --- | --- | --- | --- | --- | --- |
| $X_{3}$ | 1 | Ind | Ind | Ind | Ind | Ind | 0.413 | 0.152 |
| $X_{4}$ | Ind | 1 | Ind | Ind | Ind | Ind | 0.216 | -0.282 |
| $X_{5}$ | Ind | Ind | 1 | Ind | 0.231 | 0.096 | -0.034 | -0.304 |
| $X_{6}$ | Ind | Ind | Ind | 1 | 0.389 | -0.153 | Ind | Ind |
| $X_{7}$ | Ind | Ind | 0.231 | 0.389 | 1 | -0.179 | 0.053 | Ind |
| $X_{8}$ | Ind | Ind | 0.096 | -0.153 | -0.179 | 1 | 0.046 | -0.105 |
| $X_{9}$ | 0.413 | 0.216 | -0.034 | Ind | 0.053 | 0.046 | 1 | -0.095 |
| $X_{10}$ | 0.152 | -0.282 | -0.304 | Ind | Ind | -0.105 | -0.095 | 1 |
|  |  |  |  |  |  |  |  |  |

**Supplementary Table 2.** Strength of missingness for each variable with missing values, evaluated by taking a missing indicator variable for the variable with missing values and estimating the AUC by regressing the missing indicator variable on the other covariates related to it being missing using BART.

|  | $X_{7}$ | $X_{8}$ | $X_{9}$ | $X_{10}$ | $Y$ |
| --- | --- | --- | --- | --- | --- |
| AUC | 0.716 | 0.828 | 0.756 | 0.796 | 0.920 |

**Supplementary Table 3**. Simulation results for $n=300$ with a combination of six scenarios (3 numbers of noise variables (10, 20, 40) $\times$ 2 percentage of missingness (30% overall missingness and 60% overall missingness)). For bootstrap imputation methods on incomplete data, we show results corresponding to the best threshold values of $\pi$ based on $F_{1}$. Results on fully observed data as well as from complete cases (CC) analyses are also shown. RR-BART results with different values of $\alpha=.05, .1, .01$ are presented. Results from using the median rule (RR-BART median) are also included.

|  | **10 useful variables and 10 noise variables** | | | | | | | | | | | | | |
| --- | --- | --- | --- | --- | --- | --- | --- | --- | --- | --- | --- | --- | --- | --- |
|  | AUC | | Precision | | | Recall | | | *F*_1_ | | | Type I error | | |
|  | **Fully observed data** | | | | | | | | | | | | | |
| BART | .51 (.45, .57) | | .79 | | | .42 | | | .53 | | | .05 | | |
| XGB | .56 (.50, .67) | | .77 | | | .53 | | | .58 | | | .04 | | |
|  | 40% missingness in *Y* and 60%  overall missingness | | | | | | | 20% missingness in *Y* and 30%  overall missingness | | | | | | |
|  | AUC | Precision | | Recall | *F*_1_ | | Type I error | AUC | | Precision | Recall | | *F*_1_ | Type I error |
| RR- BART  $\alpha=.05$ | .36 (.30, .42) | .79 | | .20 | .34 | | .04 | .52 (.46, .58) | | .79 | .40 | | .51 | .03 |
| RR- BART  $\alpha=.1$ | .52 (.46, .58) | .91 | | .40 | .54 | | .11 | .56 (.50, .62) | | .81 | .48 | | .58 | .13 |
|  |  |  | |  |  | |  |  | |  |  | |  |  |
| RR- BART  $\alpha=.01$ | .32 (.26, .38) | .98 | | .13 | .29 | | .04 | .46 (.40, .52) | | .94 | .27 | | .41 | .04 |
| RR-BART (median) | .31 (.25, .37) | .54 | | .24 | .31 | | .10 | .45 (.39, .51) | | .56 | .43 | | .47 | .06 |
| BI-BART $\pi=.1$ | .37 (.31, .43) | .32 | | .41 | .38 | | .05 | .50 (.44, .56) | | .50 | .55 | | .52 | .04 |
| BI-XGB $\pi=.2$ | .50 (.44, .56) | .48 | | .52 | .50 | | .05 | .56 (.50, .63) | | .52 | .58 | | .55 | .05 |
| MIA-BART (Impute missing $Y$) | .29 (.23, .35) | .72 | | .12 | .27 | | .05 | .45 (.39, .52) | | .72 | .32 | | .44 | .06 |
| MIA-BART (Exclude missing $Y$) | .27 (.21, .33) | .71 | | .11 | .25 | | .05 | .43 (.37, .50) | | .70 | .31 | | .42 | .05 |
| MIA-XGB (Impute missing $Y$) | .42 (.35, .49) | .41 | | .45 | .43 | | .06 | .50 (.44, .57) | | .45 | .51 | | .48 | .06 |
| MIA-XGB (Exclude missing $Y$) | .40 (.33, .47) | .39 | | .43 | .41 | | .05 | .48 (.42, .55) | | .41 | .54 | | .46 | .04 |
| BART CC | .29 (.20, .38) | .24 | | .40 | .27 | | .06 | .53 (.46, .60) | | .53 | .58 | | .55 | .04 |
| XGB CC | .48 (.49, .56) | .45 | | .50 | .48 | | .05 | .52 (.45, .59) | | .48 | .54 | | .51 | .06 |
|  | **10 useful variables and 20 noise variables** | | | | | | | | | | | | | |
|  | AUC | | Precision | | | Recall | | | *F*_1_ | | | Type I error | | |
|  | **Fully observed data** | | | | | | | | | | | | | |
| BART | .53 (.47, .59) | | .81 | | | .44 | | | .55 | | | .08 | | |
| XGB | .59 (.53, .70) | | .80 | | | .56 | | | .61 | | | .04 | | |
|  | 40% missingness in *Y* and 60%  overall missingness | | | | | | | 20% missingness in *Y* and 30%  overall missingness | | | | | | |
|  | AUC | Precision | | Recall | *F*_1_ | | Type I error | AUC | | Precision | Recall | | *F*_1_ | Type I error |
| RR- BART  $\alpha=.05$ | .43 (.37, .49) | .95 | | .28 | .42 | | .09 | .54 (.48, .60) | | .86 | .38 | | .52 | .03 |
| RR- BART  $\alpha=.1$ | .45 (.39, .51) | .68 | | .33 | .46 | | .11 | .57 (.51, .63) | | .67 | .49 | | .56 | .08 |
| RR- BART  $\alpha=.01$ | .25 (.19, .31) | .87 | | .09 | .24 | | .04 | .39 (.33, .45) | | .88 | .24 | | .38 | .03 |
| RR-BART (median) | .36 (.30, 42) | .51 | | .32 | .38 | | .08 | .46 (.40, 52) | | .58 | .44 | | .48 | .06 |
| BI-BART $\pi=.1$ | .43 (.37, .49) | .40 | | .48 | .45 | | .08 | .52 (.46, .58) | | .52 | .57 | | .54 | .06 |
| BI-XGB $\pi=.2$ | .53 (.47, .59) | .51 | | .55 | .53 | | .07 | .59 (.53, .66) | | .55 | .61 | | .58 | .06 |
| MIA-BART (Impute missing $Y$) | .36 (.31, .42) | .88 | | .26 | .36 | | .07 | .47 (.41, .54) | | .80 | .33 | | .46 | .02 |
| MIA-BART (Exclude missing $Y$) | .34 (.27, .41) | .85 | | .24 | .34 | | .08 | .45 (.38, .52) | | .78 | .31 | | .44 | .03 |
| MIA-XGB (Impute missing $Y$) | .47 (.40, .52) | .44 | | .50 | .46 | | .06 | .52 (.46, .59) | | .47 | .56 | | .52 | .05 |
| MIA-XGB (Exclude missing $Y$) | .45 (.37, .52) | .42 | | .48 | .44 | | .07 | .50 (.43, .57) | | .44 | .55 | | .50 | .07 |
| BART CC | .39 (.30, .48) | .34 | | .50 | .37 | | .11 | .47 (.39, .55) | | .45 | .50 | | .47 | .09 |
| XGB CC | .50 (.41, .53) | .43 | | .53 | .49 | | .10 | .51 (.43, .59) | | .50 | .55 | | .52 | .09 |
|  | **10 useful variables and 40 noise variables** | | | | | | | | | | | | | |
|  | AUC | | Precision | | | Recall | | | *F*_1_ | | | Type I error | | |
|  | **Fully observed data** | | | | | | | | | | | | | |
| BART | .58 (.52, .64) | | .86 | | | .48 | | | .59 | | | .05 | | |
| XGB | .62 (.56, .68) | | .83 | | | .57 | | | .64 | | | .04 | | |
|  | 40% missingness in *Y* and 60%  overall missingness | | | | | | | 20% missingness in *Y* and 30%  overall missingness | | | | | | |
|  | AUC | Precision | | Recall | *F*_1_ | | Type I error | AUC | | Precision | Recall | | *F*_1_ | Type I error |
| RR- BART  $\alpha=.05$ | .52 (.46, .58) | .51 | | .56 | .54 | | .13 | .55 (.49, .61) | | .55 | .59 | | .57 | .08 |
| RR- BART  $\alpha=.1$ | .56 (.50, .62) | .41 | | .66 | .59 | | .19 | .59 (.53, .64) | | .42 | .75 | | .61 | .14 |
| RR- BART  $\alpha=.01$ | .46 (.50, .52) | .63 | | .43 | .48 | | .05 | .53 (.47, .58) | | .65 | .51 | | .55 | .04 |
| RR-BART (median) | .48 (.42, .54) | .60 | | .45 | .52 | | .15 | .52 (.46, .58) | | .62 | .48 | | .54 | .07 |
| BI-BART $\pi=.2$ | .54 (.48, .60) | .53 | | .59 | .56 | | .12 | .57 (.51, .63) | | .57 | .62 | | .59 | .06 |
| BI-XGB $\pi=.3$ | .55 (.49, .61) | .53 | | .57 | .55 | | .11 | .61 (.55, .68) | | .57 | .63 | | .60 | .08 |
| MIA-BART (Impute missing $Y$) | .46 (.40, .52) | .45 | | .50 | .48 | | .10 | .50 (.44, .56) | | .49 | .53 | | .52 | .09 |
| MIA-BART (Exclude missing $Y$) | .44 (.37, .51) | .43 | | .49 | .46 | | .08 | .48 (.42, .54) | | .46 | .52 | | .50 | .06 |
| MIA-XGB (Impute missing $Y$) | .50 (.44, .56) | .48 | | .52 | .50 | | .07 | .55 (.49, .62) | | .53 | .57 | | .56 | .02 |
| MIA-XGB (Exclude missing $Y$) | .48 (.41, .55) | .45 | | .51 | .48 | | .09 | .53 (.47, .60) | | .51 | .55 | | .53 | .04 |
| BART CC | .50 (.41, .59) | .45 | | .51 | .48 | | .15 | .52 (.44, .60) | | .50 | .55 | | .52 | .10 |
| XGB CC | .52 (.43, .55) | .46 | | .55 | .51 | | .12 | .53 (.45, .61) | | .52 | .57 | | .54 | .11 |

**Supplementary Table 3**. Simulation results for $n=650$ with a combination of six scenarios (3 numbers of noise variables (10, 20, 40) $\times$ 2 percentage of missingness (30% overall missingness and 60% overall missingness)). For bootstrap imputation methods on incomplete data, we show results corresponding to the best threshold values of $\pi$ based on $F_{1}$. Results on fully observed data as well as from complete cases (CC) analyses are also shown. RR-BART results with different values of $\alpha=.05, .1, .01$ are presented. Results from using the median rule (RR-BART median) are also included.

|  | **10 useful variables and 10 noise variables** | | | | | | | | | | | | | |
| --- | --- | --- | --- | --- | --- | --- | --- | --- | --- | --- | --- | --- | --- | --- |
|  | AUC | | Precision | | | Recall | | | *F*_1_ | | | Type I error | | |
|  | **Fully observed data** | | | | | | | | | | | | | |
| BART | .53 (.48, .58) | | .72 | | | .45 | | | .56 | | | .04 | | |
| XGB | .66 (.61, .71) | | .78 | | | .61 | | | .68 | | | .05 | | |
|  | 40% missingness in *Y* and 60%  overall missingness | | | | | | | 20% missingness in *Y* and 30%  overall missingness | | | | | | |
|  | AUC | Precision | | Recall | *F*_1_ | | Type I error | AUC | | Precision | Recall | | *F*_1_ | Type I error |
| RR- BART  $\alpha=.05$ | .47 (.42, .52) | .83 | | .36 | .49 | | .05 | .60 (.55, .65) | | .88 | .56 | | .63 | .08 |
| RR- BART  $\alpha=.1$ | .55 (.50, .60) | .81 | | .46 | .58 | | .10 | .77 (.72, .82) | | .87 | .74 | | .79 | .15 |
| RR- BART  $\alpha=.01$ | .37 (.32, .42) | .87 | | .24 | .38 | | .04 | .59 (.54, .64) | | .92 | .48 | | .60 | .03 |
| RR-BART (median) | .42 (.37, .47) | .66 | | .37 | .44 | | .08 | .52 (.47, .57) | | .65 | .46 | | .54 | .06 |
| BI-BART $\pi=.1$ | .52 (.47, .57) | .74 | | .39 | .52 | | .06 | .61 (.56, .66) | | .83 | .57 | | .63 | .03 |
| BI-XGB $\pi=.2$ | .60 (.55, .65) | .80 | | .56 | .59 | | .04 | .67 (.62, .72) | | .85 | .60 | | .67 | .04 |
| MIA-BART (Impute missing $Y$) | .41 (.36, .46) | .77 | | .30 | .43 | | .08 | .54 (.47, .61) | | .77 | .46 | | .54 | .03 |
| MIA-BART (Exclude missing $Y$) | .39 (.32, .46) | .75 | | .28 | .41 | | .07 | .51 (.44, .58) | | .74 | .43 | | .51 | .04 |
| MIA-XGB (Impute missing $Y$) | .53 (.48, .58) | .77 | | .49 | .54 | | .05 | .60 (.55, .70) | | .78 | .53 | | .59 | .04 |
| MIA-XGB (Exclude missing $Y$) | .50 (.44, .56) | .74 | | .46 | .51 | | .06 | .58 (.52, .69) | | .75 | .45 | | .55 | .03 |
| BART CC | .48 (.41, .55) | .66 | | .44 | .48 | | .06 | .56 (.49, .63) | | .81 | .45 | | .57 | .03 |
| XGB CC | .56 (.49, .64) | .72 | | .49 | .56 | | .08 | .57 (.50, .64) | | .80 | .48 | | .58 | .04 |
|  | **10 useful variables and 20 noise variables** | | | | | | | | | | | | | |
|  | AUC | | Precision | | | Recall | | | *F*_1_ | | | Type I error | | |
|  | **Fully observed data** | | | | | | | | | | | | | |
| BART | .63 (.58, .68) | | .82 | | | .55 | | | .66 | | | .03 | | |
| XGB | .70 (.65, .75) | | .82 | | | .65 | | | .72 | | | .04 | | |
|  | 40% missingness in *Y* and 60%  overall missingness | | | | | | | 20% missingness in *Y* and 30%  overall missingness | | | | | | |
|  | AUC | Precision | | Recall | *F*_1_ | | Type I error | AUC | | Precision | Recall | | *F*_1_ | Type I error |
| RR- BART  $\alpha=.05$ | .57 (.52, .62) | .85 | | .47 | .59 | | .05 | .62 (.57, .67) | | .66 | .65 | | .65 | .08 |
| RR- BART  $\alpha=.1$ | .62 (.57, .67) | .80 | | .57 | .65 | | .08 | .64 (.59, .69) | | .61 | .75 | | .67 | .10 |
| RR- BART  $\alpha=.01$ | .50 (.45, .55) | .93 | | .30 | .45 | | .05 | .61 (.56, .66) | | .78 | .54 | | .63 | .04 |
| RR-BART (median) | .52 (.47, .57) | .70 | | .46 | .54 | | .07 | .58 (.53, .63) | | .71 | .48 | | .59 | .08 |
| BI-BART $\pi=.1$ | .62 (.57, .67) | .84 | | .49 | .62 | | .06 | .63 (.58, .68) | | .63 | .67 | | .65 | .05 |
| BI-XGB $\pi=.2$ | .64 (.59, .69) | .83 | | .60 | .63 | | .05 | .69 (.64, .74) | | .65 | .70 | | .69 | .06 |
| MIA-BART (Impute missing $Y$) | .50 (.45, .65) | .80 | | .40 | .52 | | .06 | .56 (.49, .63) | | .57 | .56 | | .56 | .06 |
| MIA-BART (Exclude missing $Y$) | .48 (.42, .54) | .77 | | .38 | .50 | | .07 | .53 (.46, .60) | | .54 | .53 | | .53 | .07 |
| MIA-XGB (Impute missing $Y$) | .57 (.52, .60) | .78 | | .56 | .56 | | .06 | .62 (.57, .72) | | .58 | .63 | | .61 | .06 |
| MIA-XGB (Exclude missing $Y$) | .55 (.49, .62) | .76 | | .54 | .54 | | .07 | .60 (.54, .71) | | .65 | .55 | | .59 | .06 |
| BART CC | .58 (.51, .65) | .76 | | .54 | .58 | | .06 | .58 (.51, .65) | | .71 | .55 | | .59 | .06 |
| XGB CC | .60 (.53, .67) | .76 | | .53 | .60 | | .07 | .59 (.52, .66) | | .72 | .58 | | .60 | .06 |
|  | **10 useful variables and 40 noise variables** | | | | | | | | | | | | | |
|  | AUC | | Precision | | | Recall | | | *F*_1_ | | | Type I error | | |
|  | **Fully observed data** | | | | | | | | | | | | | |
| BART | .68 (.63, .73) | | .87 | | | .59 | | | .71 | | | .04 | | |
| XGB | .72 (.67, .77) | | .84 | | | .67 | | | .74 | | | .04 | | |
|  | 40% missingness in *Y* and 60%  overall missingness | | | | | | | 20% missingness in *Y* and 30%  overall missingness | | | | | | |
|  | AUC | Precision | | Recall | *F*_1_ | | Type I error | AUC | | Precision | Recall | | *F*_1_ | Type I error |
| RR- BART  $\alpha=.05$ | .62 (.57, .67) | .62 | | .66 | .64 | | .06 | .65 (.60, .70) | | .66 | .69 | | .68 | .04 |
| RR- BART  $\alpha=.1$ | .67 (.62, .72) | .57 | | .76 | .69 | | .09 | .67 (.62, .72) | | .61 | .79 | | .71 | .08 |
| RR- BART  $\alpha=.01$ | .52 (.47, .57) | .70 | | .51 | .49 | | .03 | .63 (.58, .68) | | .75 | .64 | | .65 | .03 |
| RR-BART (median) | .54 (.49, .59) | .64 | | .46 | .56 | | .08 | .57 (.52, .62) | | .72 | .48 | | .60 | .08 |
| BI-BART $\pi=.2$ | .64 (.59, .69) | .64 | | .69 | .67 | | .07 | .67 (.62, .72) | | .67 | .72 | | .69 | .04 |
| BI-XGB $\pi=.3$ | .65 (.60, .70) | .63 | | .67 | .65 | | .06 | .71 (.66, .77) | | .67 | .73 | | .71 | .05 |
| MIA-BART (Impute missing $Y$) | .55 (.50, .60) | .55 | | .60 | .57 | | .07 | .60 (.53, .67) | | .61 | .62 | | .62 | .05 |
| MIA-BART (Exclude missing $Y$) | .52 (.45, .59) | .50 | | .59 | .55 | | .06 | .58 (.51, .65) | | .59 | .60 | | .60 | .04 |
| MIA-XGB (Impute missing $Y$) | .59 (.54, .63) | .58 | | .60 | .59 | | .08 | .66 (.61, .76) | | .62 | .67 | | .65 | .04 |
| MIA-XGB (Exclude missing $Y$) | .56 (.49, .63) | .55 | | .58 | .56 | | .07 | .64 (.57, .72) | | .60 | .65 | | .63 | .05 |
| BART CC | .62 (.55, .69) | .61 | | .65 | .63 | | .05 | .62 (.55, .69) | | .61 | .65 | | .63 | .05 |
| XGB CC | .63 (.56, .70) | .62 | | .68 | .65 | | .08 | .63 (.56, .70) | | .62 | .68 | | .65 | .08 |

**Supplementary Table 4**. Simulation results for $n=1000$ with a combination of six scenarios (3 numbers of noise variables (10, 20, 40) $\times$ 2 percentage of missingness (30% overall missingness and 60% overall missingness)). For bootstrap imputation methods on incomplete data, we show results corresponding to the best threshold values of $\pi$ based on $F_{1}$. Results on fully observed data as well as from complete cases (CC) analyses are also shown. RR-BART results with different values of $\alpha=.05, .1, .01$ are presented. Results from using the median rule (RR-BART median) are also included.

|  | **10 useful variables and 10 noise variables** | | | | | | | | | | | | | |
| --- | --- | --- | --- | --- | --- | --- | --- | --- | --- | --- | --- | --- | --- | --- |
|  | AUC | | Precision | | | Recall | | | *F*_1_ | | | Type I error | | |
|  | **Fully observed data** | | | | | | | | | | | | | |
| BART | .76 (.72, .80) | | .96 | | | .57 | | | .76 | | | .02 | | |
| XGB | .80 (.76, .84) | | .92 | | | .61 | | | .78 | | | .03 | | |
|  | 40% missingness in *Y* and 60%  overall missingness | | | | | | | 20% missingness in *Y* and 30%  overall missingness | | | | | | |
|  | AUC | Precision | | Recall | *F*_1_ | | Type I error | AUC | | Precision | Recall | | *F*_1_ | Type I error |
| RR- BART  $\alpha=.05$ | .66 (.62, .70) | .95 | | .48 | .64 | | .05 | .76 (.72, .80) | | .95 | .64 | | .76 | .02 |
| RR- BART  $\alpha=.1$ | .76 (.72, .80) | .92 | | .58 | .70 | | .09 | .79 (.75, .83) | | .97 | .71 | | .80 | .07 |
| RR- BART  $\alpha=.01$ | .56 (.52, .60) | .98 | | .33 | .47 | | .02 | .69 (.65, .73) | | .99 | .51 | | .65 | .01 |
| RR-BART (median) | .51 (.47, .55) | .64 | | .44 | .53 | | .09 | .64 (.60, .68) | | .73 | .56 | | .66 | .08 |
| BI-BART $\pi=.1$ | .69 (.64, .74) | .89 | | .52 | .71 | | .03 | .77 (.73, .81) | | .95 | .61 | | .78 | .02 |
| BI-XGB $\pi=.2$ | .74 (.69, .79) | .84 | | .60 | .74 | | .04 | .78 (.73, 83) | | .94 | .62 | | .78 | .02 |
| MIA-BART (Impute missing $Y$) | .62 (.57, .67) | .82 | | .45 | .68 | | .04 | .70 (.66, .73) | | .90 | .51 | | .71 | .03 |
| MIA-BART (Exclude missing $Y$) | .60 (.55, .65) | .80 | | .42 | .66 | | .05 | .68 (.64, .71) | | .88 | .50 | | .79 | .04 |
| MIA-XGB (Impute missing $Y$) | .67 (.62, .72) | .78 | | .55 | .67 | | .05 | .71 (.66, 76) | | .90 | .52 | | .71 | .03 |
| MIA-XGB (Exclude missing $Y$) | .64 (.59, .69) | .75 | | .53 | .64 | | .05 | .69 (.64, 74) | | .88 | .50 | | .69 | .04 |
| BART CC | .56 (.49, .63) | .95 | | .40 | .58 | | .05 | .63 (.56, .70) | | .88 | .50 | | .65 | .03 |
| XGB CC | .67 (.60, .74) | .90 | | .54 | .71 | | .04 | .71 (.65, .77) | | .92 | .56 | | .73 | .03 |
|  | **10 useful variables and 20 noise variables** | | | | | | | | | | | | | |
|  | AUC | | Precision | | | Recall | | | *F*_1_ | | | Type I error | | |
|  | **Fully observed data** | | | | | | | | | | | | | |
| BART | .82 (.78, .86) | | .94 | | | .67 | | | .82 | | | .01 | | |
| XGB | .83 (.79, .87) | | .90 | | | .71 | | | .81 | | | .04 | | |
|  | 40% missingness in *Y* and 60%  overall missingness | | | | | | | 20% missingness in *Y* and 30%  overall missingness | | | | | | |
|  | AUC | Precision | | Recall | *F*_1_ | | Type I error | AUC | | Precision | Recall | | *F*_1_ | Type I error |
| RR- BART  $\alpha=.05$ | .72 (.68, .76) | .88 | | .59 | .70 | | .05 | .80 (.76, .84) | | .89 | .74 | | .80 | .02 |
| RR- BART  $\alpha=.1$ | .75 (.71, .79) | .80 | | .68 | .73 | | .09 | .78 (.74, .82) | | .69 | .78 | | .73 | .06 |
| RR- BART  $\alpha=.01$ | .65 (.61, .69) | .93 | | .46 | .61 | | .02 | .73 (.69, .77) | | .87 | .61 | | .71 | .02 |
| RR-BART (median) | .63 (.59, .67) | .72 | | .54 | .65 | | .09 | .66 (.62, .70) | | .76 | .59 | | .68 | .10 |
| BI-BART $\pi=.1$ | .73 (.68, .78) | .86 | | .62 | .75 | | .04 | .80 (.76, .84) | | .93 | .69 | | .82 | .01 |
| BI-XGB $\pi=.2$ | .78 (.73, .83) | .88 | | .63 | .78 | | .03 | .81 (.76, 86) | | .94 | .72 | | .81 | .03 |
| MIA-BART (Impute missing $Y$) | .65 (.61, .69) | .81 | | .52 | .62 | | .04 | .78 (.73, .83) | | .83 | .71 | | .76 | .03 |
| MIA-BART (Exclude missing $Y$) | .62 (.58, .66) | .78 | | .50 | .60 | | .03 | .75 (.70, .80) | | .80 | .68 | | .73 | .04 |
| MIA-XGB (Impute missing $Y$) | .71 (.66, .76) | .82 | | .55 | .72 | | .04 | .73 (.68, 78) | | .88 | .66 | | .75 | .04 |
| MIA-XGB (Exclude missing $Y$) | .69 (.64, .74) | .80 | | .54 | .70 | | .03 | .70 (.65, 75) | | .85 | .64 | | .72 | .05 |
| BART CC | .60 (.53, .67) | .93 | | .45 | .62 | | .05 | .67 (.60, .74) | | .92 | .54 | | .69 | .04 |
| XGB CC | .70 (.63, .77) | .94 | | .58 | .74 | | .03 | .74 (.68, .80) | | .90 | .66 | | .77 | .04 |
|  | **10 useful variables and 40 noise variables** | | | | | | | | | | | | | |
|  | AUC | | Precision | | | Recall | | | *F*_1_ | | | Type I error | | |
|  | **Fully observed data** | | | | | | | | | | | | | |
| BART | .92 (.88, .96) | | 1 | | | .87 | | | .93 | | | 0 | | |
| XGB | .88 (.84, .92) | | .93 | | | .81 | | | .86 | | | .02 | | |
|  | 40% missingness in *Y* and 60%  overall missingness | | | | | | | 20% missingness in *Y* and 30%  overall missingness | | | | | | |
|  | AUC | Precision | | Recall | *F*_1_ | | Type I error | AUC | | Precision | Recall | | *F*_1_ | Type I error |
| RR- BART  $\alpha=.05$ | .82 (.78, .86) | .87 | | .80 | .83 | | .01 | .86 (.82, .90) | | .91 | .84 | | .87 | .02 |
| RR- BART  $\alpha=.1$ | .84 (.80, .88) | .79 | | .90 | .85 | | .06 | .84 (.80, .88) | | .81 | .88 | | .85 | .06 |
| RR- BART  $\alpha=.01$ | .74 (.70, .78) | .90 | | .71 | .76 | | .02 | .79 (.75, .83) | | .92 | .72 | | .80 | .02 |
| RR-BART (median) | .69 (.65, .73) | .78 | | .62 | .70 | | .08 | .73 (.69, .77) | | .80 | .68 | | .74 | .07 |
| BI-BART $\pi=.1$ | .83 (.78, .88) | .87 | | .82 | .85 | | .01 | .87 (.83, .91) | | .91 | .87 | | .89 | .01 |
| BI-XGB $\pi=.3$ | .82 (.77, .87) | .83 | | .83 | .83 | | .03 | .85 (.80, 90) | | .99 | .76 | | .85 | .02 |
| MIA-BART (Impute missing $Y$) | .75 (.72, .79) | .80 | | .75 | .77 | | .04 | .78 (.74, .82) | | .83 | .77 | | .79 | .03 |
| MIA-BART (Exclude missing $Y$) | .73 (.66, .78) | .78 | | .70 | .70 | | .05 | .76 (.70, .82) | | .81 | .74 | | .78 | .04 |
| MIA-XGB (Impute missing $Y$) | .74 (.70, .78) | .78 | | .70 | .74 | | .05 | .77 (.73, .82) | | .83 | .76 | | .79 | .03 |
| MIA-XGB (Exclude missing $Y$) | .71 (.65, .77) | .75 | | .71 | .73 | | .04 | .73 (.67, .79) | | .68 | .73 | | .75 | .06 |
| BART CC | .70 (.63, .77) | .90 | | .60 | .72 | | .03 | .73 (.67, .79) | | .92 | .64 | | .75 | .03 |
| XGB CC | .73 (.66, .80) | .90 | | .68 | .77 | | .04 | .76 (.70, .82) | | .93 | .71 | | .80 | .03 |

**Supplementary Table 5**. Simulation results for $n=5000$ with a combination of six scenarios (3 numbers of noise variables (10, 20, 40) $\times$ 2 percentage of missingness (30% overall missingness and 60% overall missingness)). For bootstrap imputation methods on incomplete data, we show results corresponding to the best threshold values of $\pi$ based on $F_{1}$. Results on fully observed data as well as from complete cases (CC) analyses are also shown. RR-BART results with different values of $\alpha=.05, .1, .01$ are presented. Results from using the median rule (RR-BART median) are also included.

|  | **10 useful variables and 10 noise variables** | | | | | | | | | | | | | |
| --- | --- | --- | --- | --- | --- | --- | --- | --- | --- | --- | --- | --- | --- | --- |
|  | AUC | | Precision | | | Recall | | | *F*_1_ | | | Type I error | | |
|  | **Fully observed data** | | | | | | | | | | | | | |
| BART | .87 (.85, .89) | | .92 | | | .88 | | | .89 | | | .04 | | |
| XGB | .86 (.84, .88) | | .90 | | | .81 | | | .84 | | | .05 | | |
|  | 40% missingness in *Y* and 60%  overall missingness | | | | | | | 20% missingness in *Y* and 30%  overall missingness | | | | | | |
|  | AUC | Precision | | Recall | *F*_1_ | | Type I error | AUC | | Precision | Recall | | *F*_1_ | Type I error |
| RR- BART  $\alpha=.05$ | .82 (.80, .84) | .85 | | .78 | .81 | | .03 | .85 (.83, .87) | | .88 | .83 | | .85 | .05 |
| RR- BART  $\alpha=.1$ | .85 (.83, .87) | .80 | | .88 | .84 | | .12 | .89 (.87, .91) | | .86 | .91 | | .88 | .10 |
| RR- BART  $\alpha=.01$ | .82 (.80, .84) | .90 | | .72 | .80 | | .04 | .82 (.80, .84) | | .90 | .74 | | .81 | .03 |
| RR-BART (median) | .73 (.71, .75) | .74 | | .66 | .72 | | .05 | .72 (70, .74) | | .72 | .68 | | .70 | .04 |
| BI-BART $\pi=.1$ | .81 (.79, .83) | .84 | | .81 | .81 | | .05 | .86 (.84, .98) | | .90 | .85 | | .88 | .05 |
| BI-XGB $\pi=.2$ | .82 (.80, .84) | .83 | | .81 | .82 | | .05 | .84 (.82, .86) | | .87 | .83 | | .85 | .05 |
| MIA-BART (Impute missing $Y$) | .75 (.73, .77) | .78 | | .66 | .76 | | .08 | .78 (.76, .80) | | .80 | .71 | | .77 | .05 |
| MIA-BART (Exclude missing $Y$) | .73 (.69, .77) | .74 | | .66 | .70 | | .06 | .74 (.71, .77) | | .74 | .71 | | .73 | .06 |
| MIA-XGB (Impute missing $Y$) | .75 (.73, .77) | .73 | | .71 | .73 | | .06 | .75 (.73, .77) | | .78 | .75 | | .77 | .04 |
| MIA-XGB (Exclude missing $Y$) | .71 (.67, .75) | .73 | | .75 | .74 | | .07 | .74 (.71, .77) | | .76 | .74 | | .75 | .04 |
| BART CC | .69 (.65, .73) | .86 | | .61 | .70 | | .03 | .74 (.71, .77) | | .91 | .67 | | .78 | .04 |
| XGB CC | .74 (.70, .78) | .90 | | .68 | .76 | | .04 | .77 (.74, .80) | | .87 | .73 | | .79 | .04 |
|  | **10 useful variables and 20 noise variables** | | | | | | | | | | | | | |
|  | AUC | | Precision | | | Recall | | | *F*_1_ | | | Type I error | | |
|  | **Fully observed data** | | | | | | | | | | | | | |
| BART | .90 (.88, .92) | | .94 | | | .90 | | | .92 | | | .03 | | |
| XGB | .89 (.87, .91) | | .93 | | | .83 | | | .87 | | | .04 | | |
|  | 40% missingness in *Y* and 60%  overall missingness | | | | | | | 20% missingness in *Y* and 30%  overall missingness | | | | | | |
|  | AUC | Precision | | Recall | *F*_1_ | | Type I error | AUC | | Precision | Recall | | *F*_1_ | Type I error |
| RR- BART  $\alpha=.05$ | .84 (.82, .86) | .86 | | .81 | .83 | | .04 | .87 (.85, .89) | | .90 | .85 | | .87 | .04 |
| RR- BART  $\alpha=.1$ | .90 (.88, .92) | .82 | | .93 | .90 | | .14 | .88 (.86, .88) | | .88 | .90. | | .89 | .10 |
| RR- BART  $\alpha=.01$ | .85 (.83, .87) | 1 | | .80 | .82 | | .02 | .83 (.81, .85) | | .98 | .79 | | .82 | .02 |
| RR-BART (median) | .76 (.74, .78) | .78 | | .64 | .74 | | .16 | .73 (.71, .75) | | .74 | .67 | | .72 | .12 |
| BI-BART $\pi=.1$ | .84 (.82, .87) | .87 | | .82 | .83 | | .04 | .89 (.87, .91) | | .92 | .88 | | .90 | .04 |
| BI-XGB $\pi=.2$ | .84 (.82, .86) | .85 | | .83 | .84 | | .04 | .86 (.84, .88) | | .89 | .85 | | .87 | .04 |
| MIA-BART (Impute missing $Y$) | .78 (.76, .80) | .80 | | .71 | .79 | | .06 | .80 (.78, .82) | | .81 | .75 | | .80 | .04 |
| MIA-BART (Exclude missing $Y$) | .75 (.71, .79) | .77 | | .70 | .73 | | .05 | .77 (.74, .80) | | .78 | .76 | | .78 | .05 |
| MIA-XGB (Impute missing $Y$) | .77 (.75, .79) | .76 | | .75 | .75 | | .05 | .78 (.76, .80) | | .80 | .78 | | .79 | .05 |
| MIA-XGB (Exclude missing $Y$) | .73 (.69, .77) | .76 | | .73 | .75 | | .06 | .76 (.73, .79) | | .79 | .76 | | .78 | .06 |
| BART CC | .72 (.68, .76) | .89 | | .63 | .74 | | .05 | .76 (.73, .79) | | .93 | .69 | | .80 | .02 |
| XGB CC | .76 (.72, .80) | .92 | | .70 | .78 | | .05 | .79 (.76, .82) | | .90 | .76 | | .81 | .05 |
|  | **10 useful variables and 40 noise variables** | | | | | | | | | | | | | |
|  | AUC | | Precision | | | Recall | | | *F*_1_ | | | Type I error | | |
|  | **Fully observed data** | | | | | | | | | | | | | |
| BART | .95 (.93, .97) | | 1 | | | .91 | | | .95 | | | .01 | | |
| XGB | .91 (.89, .93) | | .95 | | | .85 | | | .89 | | | .02 | | |
|  | 40% missingness in *Y* and 60%  overall missingness | | | | | | | 20% missingness in *Y* and 30%  overall missingness | | | | | | |
|  | AUC | Precision | | Recall | *F*_1_ | | Type I error | AUC | | Precision | Recall | | *F*_1_ | Type I error |
| RR- BART  $\alpha=.05$ | .86 (.84, .88) | .90 | | .83 | .87 | | .02 | .89 (.87, .91) | | .92 | .88 | | .90 | .01 |
| RR- BART  $\alpha=.1$ | .88 (.86, .90) | .86 | | .93 | .89 | | .10 | .93 (.91, .95) | | .88 | .95 | | .92 | .08 |
| RR- BART  $\alpha=.01$ | .87 (.85, .89) | .92 | | .78 | .86 | | .03 | .86 (.84, .88) | | .95 | .78 | | .85 | .02 |
| RR-BART (median) | .76 (.74, .78) | .78 | | .68 | .74 | | .08 | .73 (.71, .75) | | .79 | .66 | | .72 | .13 |
| BI-BART $\pi=.1$ | .87 (.85, .89) | .91 | | .85 | .88 | | .03 | .91 (.89,.93) | | .94 | .90 | | .92 | .02 |
| BI-XGB $\pi=.3$ | .86 (.84, .88) | .87 | | .86 | .86 | | .02 | .88 (.86, .90) | | .91 | .88 | | .89 | .03 |
| MIA-BART (Impute missing $Y$) | .80 (.78, .82) | .82 | | .74 | .81 | | .03 | .82 (.80, .84) | | .83 | .81 | | .82 | .03 |
| MIA-BART (Exclude missing $Y$) | .77 (.73, .81) | .80 | | .72 | .77 | | .04 | .79 (.76, .82) | | .80 | .78 | | .80 | .04 |
| MIA-XGB (Impute missing $Y$) | .79 (.77, .81) | .80 | | .78 | .79 | | .04 | .81 (.79, .81) | | .83 | .80 | | .82 | .04 |
| MIA-XGB (Exclude missing $Y$) | .76 (.72, .80) | .78 | | .76 | .77 | | .05 | .78 (.75, .81) | | .81 | .78 | | .80 | .05 |
| BART CC | .74 (.70, .78) | .91 | | .65 | .76 | | .04 | .78 (.75, .81) | | .93 | .69 | | .80 | .02 |
| XGB CC | .78 (.74, .82) | .94 | | .71 | .80 | | .04 | .81 (.78, .84) | | .94 | .77 | | .84 | .04 |

**Supplementary Table 6.** Summary statistics for the minimum average variable importance proportion (VIP) scores across 250 data replications for the simulation scenario with sample size $n=1000.$ When there are no noise predictors (left panel), the minimum average VIP is considerably further away from 0. By comparison, when there are noise predictors (right panel), the minimum average VIP is close to 0. Q1= first quartile; Q3 = third quartile.

|  | 10 useful variables and 0 noise variables | | | | | |  | 10 useful variables and 40 noise variables | | | | | |  |
| --- | --- | --- | --- | --- | --- | --- | --- | --- | --- | --- | --- | --- | --- | --- |
|  | Min | Q1 | Median | Mean | Q3 | Max |  | Min | Q1 | Median | Mean | Q3 | Max | |
| 60% overall missingness | 0.085 | 0.088 | 0.091 | 0.091 | 0.093 | 0.098 |  | 0 | 0.004 | 0.007 | 0.007 | 0.009 | 0.014 | |
| 30% overall missingness | 0.075 | 0.078 | 0.079 | 0.080 | 0.082 | 0.089 |  | 0 | 0.003 | 0.004 | 0.005 | 0.007 | 0.013 | |

**Supplementary Table 7**. Simulation results for $n=1000$ with the extreme case where there are no noise variables. For bootstrap imputation methods on incomplete data, we show results corresponding to the best threshold values of $\pi$ based on $F_{1}$. Results on fully observed data (FOD) as well as from complete cases (CC) analyses are also shown. The 95% confidence interval of AUC was calculated from 100 data repetitions and are presented in parentheses.

|  | AUC | | Precision | | | | Recall | | | *F*_1_ | | | Type I error | | |
| --- | --- | --- | --- | --- | --- | --- | --- | --- | --- | --- | --- | --- | --- | --- | --- |
|  | **Fully observed data** | | | | | | | | | | | | | | |
| BART | .74 (.68, .80) | | 1 | | | | .62 | | | .70 | | | NA | | |
| XGB | .75 (.69, .81) | | 1 | | | | .61 | | | .69 | | | NA | | |
|  | 40% missingness in *Y* and 60% overall missingness | | | | | | |  | 20% missingness in *Y* and 30% overall missingness | | | | | | |
|  | AUC | Precision | | Recall | *F*_1_ | Type I error | |  | AUC | | Precision | Recall | | *F*_1_ | Type I error |
| RR- BART $\alpha=.05$ | .73 (.67, .79) | 1 | | .36 | .48 | NA | |  | .77 (.72, .82) | | 1 | .51 | | .67 | NA |
| RR-BART (all selected) | .97 (.95, .99) | 1 | | 1 | 1 | NA | |  | .98 (.96, .99) | | 1 | 1 | | 1 | NA |
| BI-BART  $\pi=.1$ | .73 (.67, .79) | 1 | | .38 | .50 | NA | |  | .75 (.70, .80) | | 1 | .50 | | .69 | NA |
| BI-XGB  $\pi=.2$ | .79 (.73, .85) | 1 | | .54 | .64 | NA | |  | .80 (.75, .85) | | 1 | .52 | | .70 | NA |
| MIA-BART (Impute missing $Y$) | .66 (.60, .72) | 1 | | .31 | .42 | NA | |  | .70 (.65, .75) | | 1 | .46 | | .60 | NA |
| MIA-BART (Exclude missing $Y$) | .63 (.56, .70) | 1 | | .25 | .40 | NA | |  | .67 (.61, .73) | | 1 | .43 | | .57 | NA |
| MIA-XGB (Impute missing $Y$) | .73 (.67, .69) | 1 | | .50 | .59 | NA | |  | .70 (.65, .75) | | 1 | .46 | | .64 | NA |
| MIA-XGB (Exclude missing $Y$) | .70 (.62, .77) | 1 | | .46 | .55 | NA | |  | .67 (.61, .73) | | 1 | .42 | | .61 | NA |
| BART CC | .50 (.43, .57) | 1 | | .15 | .35 | NA | |  | .54 (.49, .59) | | 1 | .16 | | .39 | NA |
| XGB CC | .55 (.48, .62) | 1 | | .18 | .38 | NA | |  | .56 (.51, .61) | | 1 | .20 | | .40 | NA |

**Supplementary Table 8.** Comparison based on mean cross-validated AUC between RR-BART $\alpha=.05$ and BI-BART with the best threshold values of $\pi$ based on $F_{1}$ among 250 replications for $n=1000$ and $n=5000$, and 1000 replications for $n=300$ and $n=650$.

|  | 40% missingness in Y and 60% overall missingness | | | 20% missingness in Y and 30% overall missingness | | |
| --- | --- | --- | --- | --- | --- | --- |
|  | RR-BART better | BI-BART better | The same variables selected | RR-BART better | BI-BART better | The same variables selected |
| $n=300$ and 10 noise variables | 453 | 501 | 46 | 512 | 446 | 42 |
| $n=300$ and 20 noise variables | 477 | 489 | 34 | 515 | 448 | 37 |
| $n=300$ and 40 noise variables | 459 | 528 | 13 | 448 | 539 | 13 |
| $n=650$ and 10 noise variables | 447 | 515 | 38 | 473 | 486 | 41 |
| $n=650$ and 20 noise variables | 444 | 529 | 27 | 483 | 499 | 18 |
| $n=650$ and 40 noise variables | 487 | 498 | 15 | 481 | 500 | 19 |
| $n=1000$ and 10 noise variables | 111 | 120 | 19 | 119 | 125 | 6 |
| $n=1000$ and 20 noise variables | 118 | 125 | 7 | 121 | 120 | 9 |
| $n=1000$ and 40 noise variables | 114 | 121 | 15 | 117 | 122 | 11 |
| $n=5000$ and 10 noise variables | 122 | 120 | 8 | 131 | 115 | 4 |
| $n=5000$ and 20 noise variables | 118 | 122 | 10 | 112 | 128 | 10 |
| $n=5000$ and 40 noise variables | 116 | 128 | 6 | 117 | 126 | 7 |

**Supplementary Table 9.** The mean and Monte Carlo error of the variable inclusion proportion (VIP) for the10 useful variables from the RR-BART method among 250 data replications. The simulation configurations are: *n*=1000, the number of noise predictors = 40, 20% missingness in *Y* and 30% overall missingness. The Monte Carlo error was estimated by the standard deviation of the estimated VIPs among 250 data replications.

|  | $X_{1}$ | $X_{2}$ | $X_{3}$ | $X_{4}$ | $X_{5}$ | $X_{6}$ | $X_{7}$ | $X_{8}$ | $X_{9}$ | $X_{10}$ |
| --- | --- | --- | --- | --- | --- | --- | --- | --- | --- | --- |
| Mean | .023 | .020 | .041 | .034 | .022 | .049 | .019 | .042 | .018 | .016 |
| Monte Carlo error | .002 | .003 | .003 | .003 | .002 | .003 | .002 | .003 | .002 | .002 |

**Supplementary Table 11.** Simulation results for RR-BART and BART-pooling based on 250 data replications. The sample size $n=1000$ and the number of noise variables = 40.

|  | 40% missingness in *Y* and 60%  overall missingness | | | | | 20% missingness in *Y* and 30%  overall missingness | | | | |
| --- | --- | --- | --- | --- | --- | --- | --- | --- | --- | --- |
|  | AUC | Precision | Recall | *F*_1_ | Type I error | AUC | Precision | Recall | *F*_1_ | Type I error |
| RR- BART  $\alpha=.05$ | .82 (.78, .86) | .87 | .80 | .83 | .01 | .86 (.82, .90) | .91 | .84 | .87 | .02 |
| BART pooling | .81 (.77, .85) | .88 | .77 | .82 | .01 | .85 (.81, .89) | .92 | .82 | .86 | .01 |

**Supplementary Table 12**. Names and definitions of 60 candidate predictors in SWAN data

| Variable name | Definition |
| --- | --- |
| APOARES | Apolipoprotein A-1, milligrams per deciliter (mg/dl) |
| BMI | Body mass index |
| BP | Current medications - blood pressures medications |
| CRP | C-reactive protein, milligrams per liter (mg/l) |
| DIABP | Diastolic blood pressure |
| DTTLIN | Daily dietary estimate (DDE) linoleic acid, grams (g) |
| E2AVE | Estradiol, picograms per milliliter (average, pg/mL) |
| EDUCATION | Education |
| GLUCOSE | Blood glucose, milligrams per deciliter (mg/dl) |
| HPBMD | Total hip bone mineral density |
| INSULIN | Current medications - insulin medications |
| LMPDAY | Last menstrual period (days since baseline) |
| LPA | Lipoprotein Lp(a), milligrams per deciliter (mg/dl) |
| PAI1 | PAI-1, nanograms per milliliter (ng/ml) |
| RACE | Race/ethnicity |
| RESTLES | Frequency of restless sleep past week |
| SHBG | Sex hormone-binding globulin, nanomolar (nM) |
| SPBMD | Total spine bone mineral density |
| SYSBP | Systolic blood pressure |
| T | Testosterone, nanograms per deciliter (ng/dL) |
| TPA | Tissue plasminogen activator, nanograms per millilter (ng/ml) |
| TRIGRES | Triglycerides, milligrams per deciliter (mg/dl) |
| WAIST | Waist circumference (cm) |
| WHRATIO | Waist to hip circumference ratio |
| AGE | Age in years |
| INCOME | Total family income |
| PREPAID | Pre-paid private insurance |
| MEDICAR | Medicare |
| MEDICAI | Medicaid |
| NOINSUR | No insurance |
| NERVOUS | Current medications - nervous condition |
| INSUEVE | Insulin ever used |
| CHLDCAR | Time spent past year caring for child |
| LGTCHOR | Time spent past year light chores |
| MODCHOR | Time spent past year moderate chores |
| PHYSWOR | Work physical compared to other women same age |
| PREPMEA | Time spent past year preparing meals |
| SPORTS | Time spent past year sport/exercise |
| VIGCHOR | Time spent past year vigorous chores |
| EMOACCO | Accomplished less past month due to emotional problem |
| EMOCARE | Activity/work less carefully past month due to emotional problem |
| EMOCTDW | Cut down on activity/work past month due to emotional problem |
| FLOWDAY | How long menstrual flow lasted past year |
| STARTDA | Menstrual flow start within 4 days of when expected past year |
| SMOKERE | Ever smoked regularly |
| WAKEARL | Wake up early past 2 weeks |
| WAKEUP | Wake up several times per night past 2 weeks |
| TRBLSLE | Trouble falling asleep past 2 weeks |
| LDLRESU | Estimated low density lipoprotein (LDL) cholesterol, milligrams per deciliter (mg/dl) |
| LPA1RES | Lipoprotein A-1, milligrams per deciliter (mg/dl) |
| ALLCALC | Diet and supplement calcium, milligrams (mg) |
| DTMANG | Manganese, milligrams (mg) |
| DTTRANS | Trans fats, grams (g) |
| DTTSFAT | Daily dietary estimate (DDE) saturated fats, grams (g) |
| DTTSWET | Daily dietary estimate (DDE) percent kilocalories (%KCAL) sweets |
| FATSRV | Servings of fats/sweets/snacks |
| FATWK | Weekly variability fat/sweet |
| STATUS | Menopausal status |
| FEELBLU | Feeling blue past 2 weeks |
| MARITALGP | Current marital status/group |


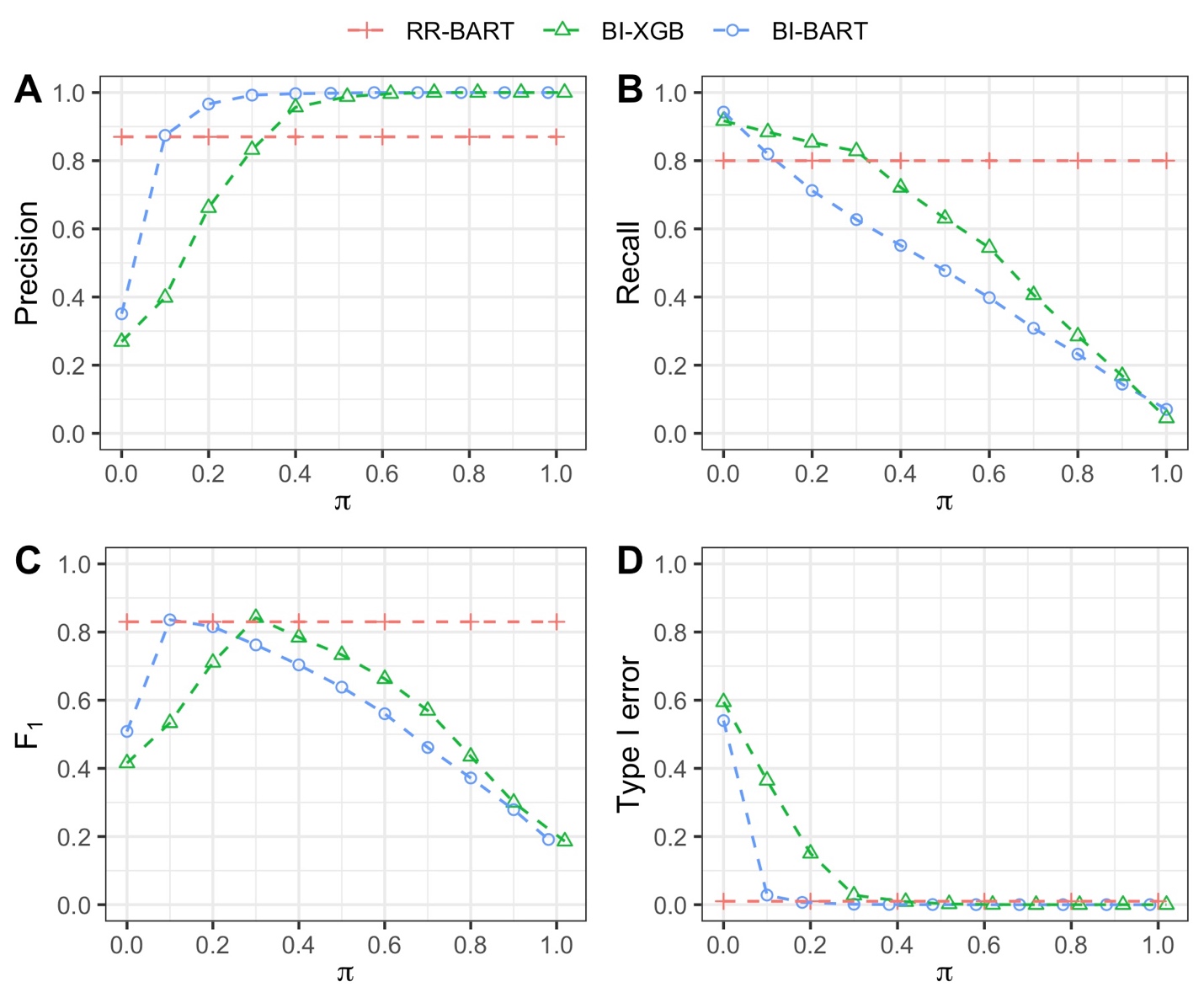


**Supplementary Figure 1**. The precision, recall, $F_{1}$ score and Type I error, for each of three methods: RR-BART, BI-BART and BI-XGB, based on 250 data replications. The performance measures for bootstrap imputation based methods BI-BART and BI-XGB vary by the threshold value of $\pi$, wheareas the performance measures of RR-BART do not. missForest was used for imputation. The sample size $n=1000.$ The proportion of missingness is 40% in the outcome $Y$and is 60% overall.


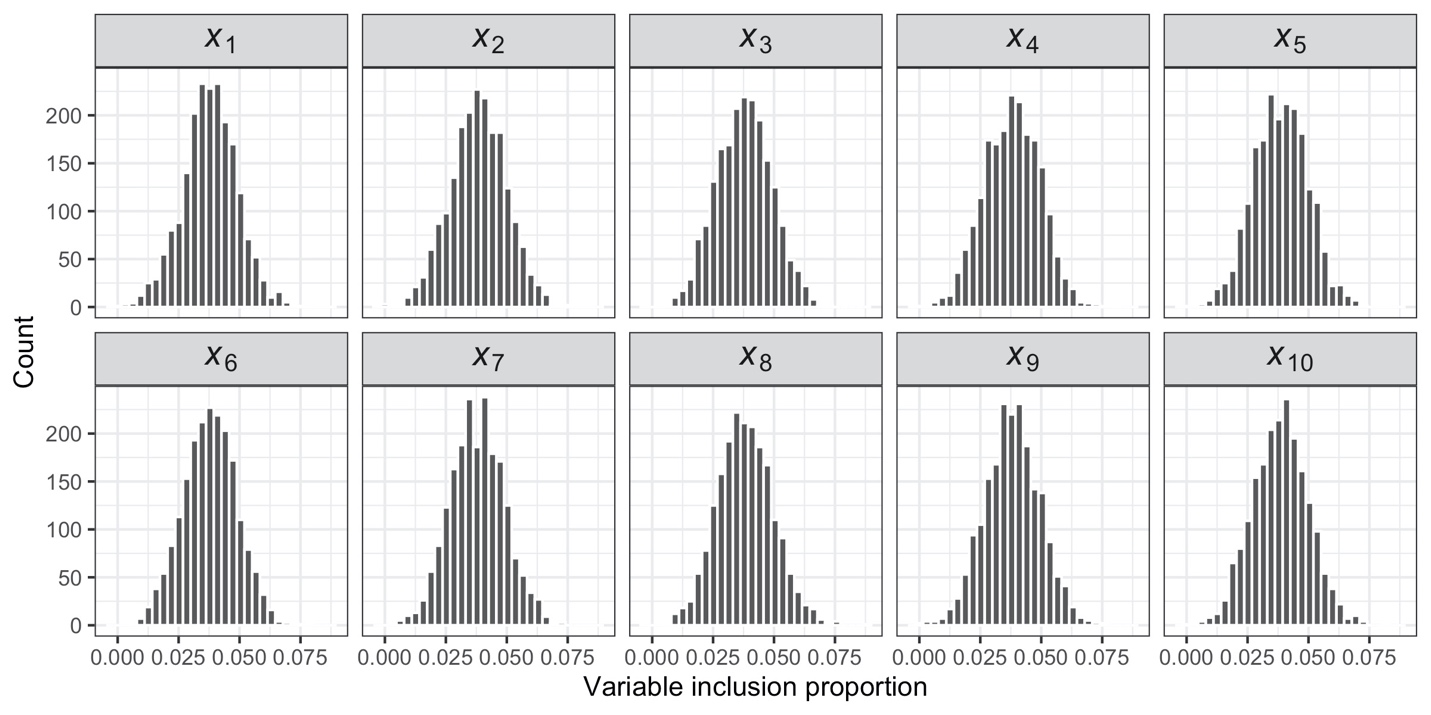


**Supplementary Figure 2**. Posterior distributions of variable selection proportions (VIP) for the 10 useful predictors with $n=1000$, 40 noise variables, 40% missingness in *Y* and 60% overall missingness. The VIP scores were calculated using BART, and missing data were imputed using missForest. When fitting BART models, we used 1100 posterior draws with the first 100 discarded as burn-in. The number of thinning was set to the default value of 1.

**Reference**

1. Schouten RM, Lugtig P, Vink G. Generating missing values for simulation purposes: a multivariate amputation procedure. *Journal of Statistical Computation and Simulation*. 2018/10/13 2018;88(15):2909-2930. doi:10.1080/00949655.2018.1491577

2. Stekhoven DJ, Bühlmann P. MissForest—non-parametric missing value imputation for mixed-type data. *Bioinformatics*. 2012;28(1):112-118. doi:10.1093/bioinformatics/btr597

3. Hu L, Joyce Lin J-Y, Ji J. Variable selection with missing data in both covariates and outcomes: Imputation and machine learning. *Statistical Methods in Medical Research*. 2021/12/01 2021;30(12):2651-2671. doi:10.1177/09622802211046385
